# Chronic Myelomonocytic Leukemia following Multicentric Castleman Disease

**DOI:** 10.1155/2018/5895903

**Published:** 2018-01-23

**Authors:** Feng Li, Xiaomei Zhang, Yanting Guo, Yuandong Zhu, Yicun Wu, Yun Ling

**Affiliations:** Department of Hematology, The First People's Hospital of Changzhou, The Third Affiliated Hospital of Soochow University, Changzhou, Jiangsu, China

## Abstract

Multicentric Castleman disease (MCD) is a rare nonmalignant lymphoproliferative disorder presenting systemic symptoms such as fever, night sweats, fatigue, anemia, effusions, and multifocal lymphadenopathy. The etiology of MCD has not been clarified to date. The coexistence of MCD with chronic myelomonocytic leukemia (CMML) has been rarely reported. Although the pathogenesis remains unclear, this association probably reflects an incidental and fortuitous finding rather than the alteration of a common pluripotent stem cell precursor. Herein, we report on one case of MCD coexisting with CMML and elucidate the underlying mechanism of pathology in some aspects.

## 1. Introduction

MCD is a rare polyclonal B lymphoproliferative disorder that straddles the intersections of hematology, oncology, rheumatology, and virology. MCD manifests with a heterogeneous group of disorders with various etiologies that demonstrate episodic systemic inflammatory symptoms, reactive proliferation of morphologically benign lymphocytes, and multiple organ system impairment [1, 2].

Chronic myelomonocytic leukemia (CMML) is a clonal stem cell disorder with features that overlap those of myelodysplastic syndromes (MDSs) and myeloproliferative neoplasms (MPNs) with a variable but overall poor prognosis. It occurs almost exclusively in the elderly with a median age at diagnosis varying between 65 and 75 years. Similar to MDS, the pathogenesis of CMML appears to be multifactorial and linked to altered genetic, molecular, microenvironmental, and immunologic state in different individuals.

Patients with MCD have a significantly high risk of developing severe pancytopenia, multiorgan failure, and lymphoma compared with healthy controls [3]. On the contrary, it is quite a rare event for MCD to progress to CMML, which belongs to different pathogenic cell types. In the present case, we first describe a MCD patient, who progressed to CMML and was sensitive to decitabine therapy.

## 2. Case Report

**Figure 1:**
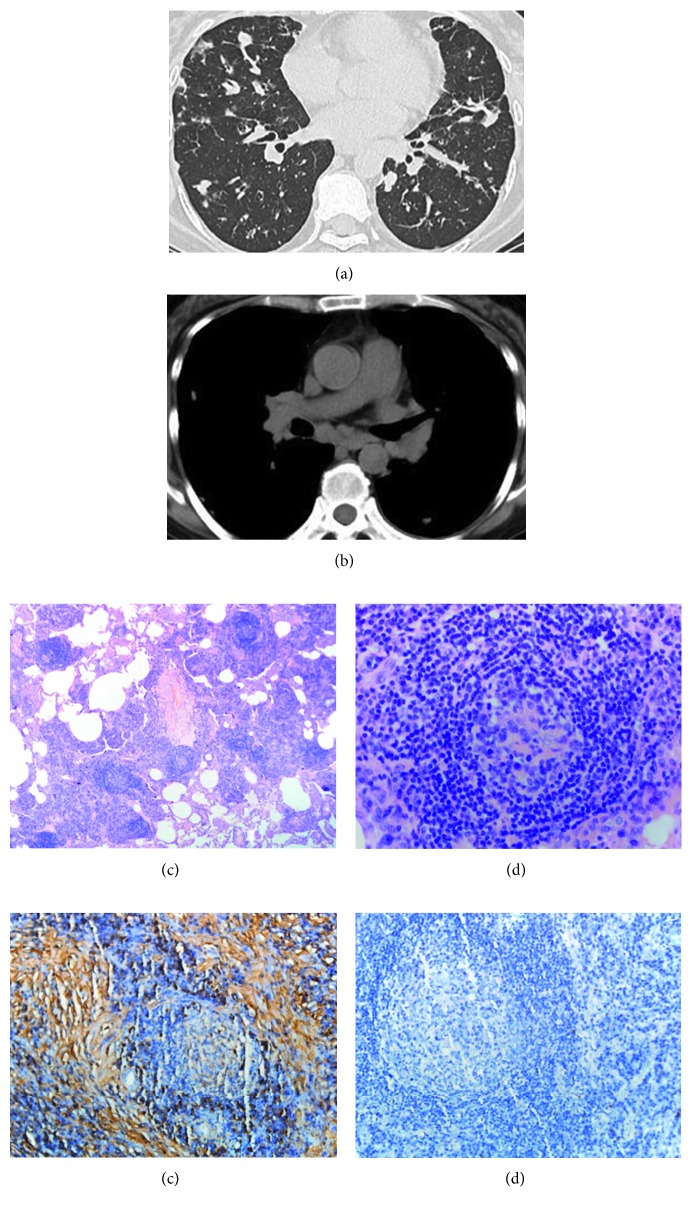
(a) and (b) HRCT of the chest at diagnosis shows bilateral centrilobular nodules, thickening of the interlobular septa, and mediastinal lymphadenopathy. (c) and (d) Histological findings of the pulmonary nodule show infiltration of polyclonal plasma cells around the bronchovascular bundles and in the interfollicular areas of the nodes (H&E, 50x and 200x). (e) and (f) Sheets of mature plasma cells are present within the interfollicular region and are highlighted by positive immunostaining of IgG- and IgG4-negative plasma cells (immunohistochemistry, 200x).

The patient, a 44-year-old woman, was admitted to our center in December 2011 suffering from cough, asthma, and asthenia. Peripheral blood studies showed severe iron-deficiency anemia, elevations of C-reactive protein, and polyclonal hypergammaglobulinemia. Computed tomogram demonstrated diffuse (lung, axillary, and mediastinal) lymphadenopathy, interstitial pneumonitis, and splenomegaly (Figures 1 and 1). Histology of left pulmonary nodule accessed by thoracic surgery revealed atretic follicles with plasmacytosis, prominent paracortical vascularity, and sinus histiocytosis with an overall appearance consistent with MCD (Figures 1 and 1). Immunohistochemistry for IgG and CD138 was positive, and IgG4 was negative (Figures 1 and 1). Based on clinical and pathological suspicion of Castleman disease, further testing revealed high interleukin-6 (IL-6) of 23.0 pg/mL and normal IgG4 of 1.28 g/L. The patient's serum tested negative for HIV, and no cytomegalovirus, Epstein–Barr virus, or human herpes virus-8 (HHV-8) replication was detected. She was diagnosed with HIV-negative and HHV-8-negative multicentric Castleman disease of plasma cell variant. She was started on monotherapy chemotherapy with low-dose cyclophosphamide, accompanied by oral steroids from March 2012.

**Figure 2:**
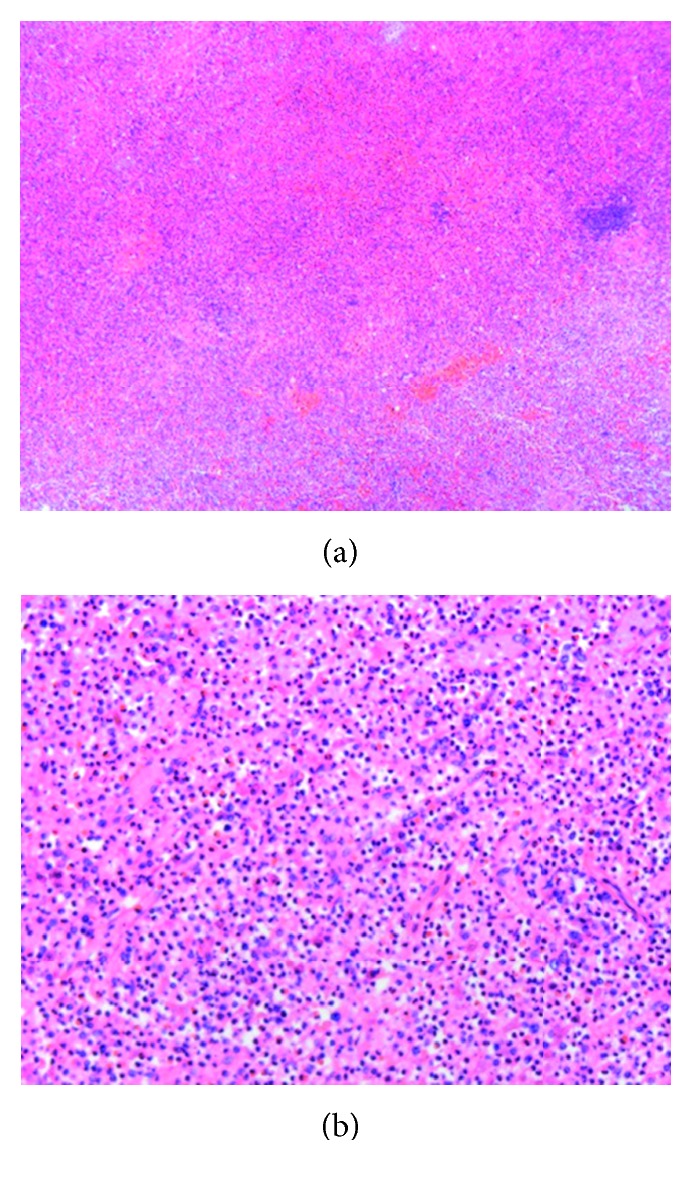
(a) and (b) Pathological section demonstrated the structural breakdown and significant hypercellularity with hyperplastic granulopoiesis (H&E, 50x and 200x).

**Figure 3:**
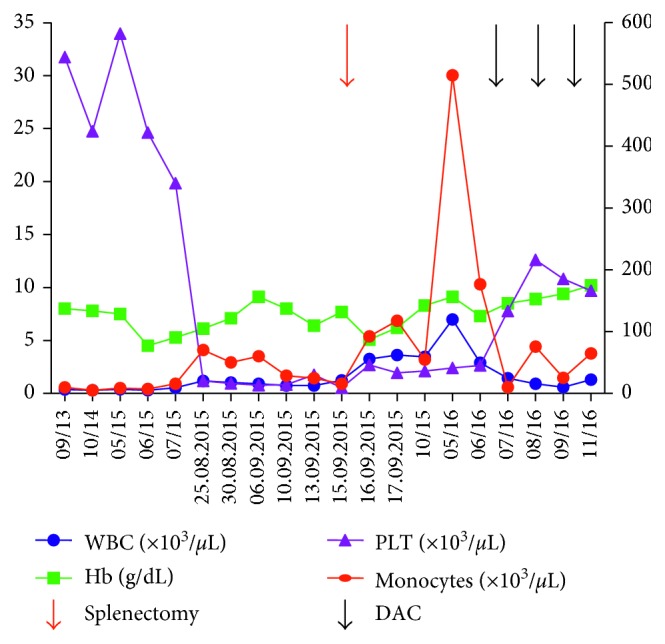
Blood counts of the patient during chronic treatment with prednisone and cyclophosphamide to MCD and conservative treatment with DAC. WBC and PLT used right-sided *y*-axis, while left-sided *y*-axis was used for Hb and monocytes.

Unfortunately, she was later readmitted to our hospital for severe abdominal pain and skin and mucous membrane bleeding in August 2015 with a history of type-II diabetes for two years. Just one month ago, she followed the doctors' advice of cessation of the cyclophosphamide for severe anemia (Hb, 4.4 g/dL). Continued monitoring of routine blood count showed normocytic normochromic anemia (Hb, 6.0–8.0 g/dL), severe thrombocytopenia (PLT, 10,000–20,000/*μ*L), and mild leukocytosis (10,000–12,000/*μ*L), while monocytes were at normal levels. Bone marrow (BM) study showed an increased number of megakaryocytes, and myeloid series hypercellularity and dysplastic change were evident. Abdominal CT showed severe splenomegaly and multifocal and wide-area infarction. Subsequently, she underwent laparoscopic splenectomy, and the pathological section demonstrated the structural breakdown and significant hypercellularity (Figures 2 and 2). In next nine months, there was a stable increase of WBC (20,000–100,000/*μ*L), especially peripheral blood monocytes (1,500–10,000/*μ*L), which was still accompanied by decreased platelet counts (15,000–60,000/*μ*L) and anemia (Hb, 6.0–8.0 g/dL). At this period, BM examination was again performed and revealed a significant increase in monocytes (12%) and prominent dysplastic changes in myeloid lineages. High expression levels of CD13, CD33, CD14, CD64, and CD56 were detected in her bone marrow cells. The karyotype was normal, and BCR/ABL fusion gene was not detected by the fluorescent in situ hybridization. At the same time, molecular studies were negative for TET2, SRSF2, ASXL1, and SETBP1 mutations, which are reported in about 90% of patients with CMML. According to World Health Organization (WHO) classification, a diagnosis of CMML and MCD, which were simultaneously present, was made. Then, she received front-line treatment with decitabine (DAC, 20 mg/m^2^ for 5 d in a 4-week cycle) for CMML. Surprisingly, after just one cycle, the WBC count had declined to 15,000/*μ*L and the Hb increased to 9.0 g/dL and PLT to 220,000/*μ*L (Figure 3). She also was recommended allogeneic hematopoietic stem cell transplant (HSCT) as the only potentially curative option.

## 3. Discussion

MCD is a rare nonmalignant lymphoproliferative disorder, and the population incidence has not been established. Little is known about the clinical abnormalities, disease associations, treatments, and outcomes. Systemic manifestations such as fever, night sweats, fatigue, anemia, and multifocal lymphadenopathy appear as a result of excessive IL-6 and other proinflammatory cytokines [2, 4]. Three treatment strategies have been used based on our understanding of MCD: anti-inflammatory and immunosuppressive therapies, cytotoxic elimination of cells responsible for hypercytokinemia, and blockade of IL-6 signaling with mAbs [5–7]. In addition, glucocorticoids have frequently been used in MCD. The response rate is usually high (60–70%), but symptoms typically recur with decreasing dose. Autologous stem cell transplantation has been successful in challenging cases [8]. Recently, one study found that multicentricity, histopathological type, and anemia are significant risk factors for shortened progression-free survival [9]. This case just only received monotherapy chemotherapy and prednisone and got long survival.

The current diagnostic criteria of CMML have recently been updated by the 2016 WHO classification. Persistent monocytosis defined both as an absolute count > 1,000/*μ*L and as a proportion > 10% of WBC count is the cornerstone of diagnosis. CMML has a tendency to transform to acute myeloid leukemia (AML). Given the overlap with MDS and MPN, management and response evaluation for patients with CMML is often extrapolated from these diseases. However, internationally validated treatment algorithms for CMML are still lacking, and treatment remains loosely codified. The hypomethylating agents (HMA) azacitidine (AZA) and DAC seem active in CMML, though they have mostly been explored retrospectively, reporting overall response rates in the 40–70% range [10, 11]. For this case, the management of cytopenias is similar to that for MDS based on the MDS-like features and had a good response for DAC.

To our knowledge, there have been some studies founding that subjects with CMML have a high prevalence of chronic inflammatory or autoimmune disorders, especially systemic vasculitis, connective tissue diseases, and immune thrombocytopenia (ITP) [12–14]. This is in line with chronic inflammation acting as a trigger and driver of clonal evolution in CMML, implying an increased risk of CMML in patients with inflammatory and/or autoimmune diseases [15]. The reasons for this increased risk are unknown but may be due to immune deregulation with aberrant immune responses and impaired tumor immune surveillance.

MCD has an inherent tendency to progress to lymphoma, and the driving molecular aberrations involved in transformation remain obscure [16, 17]. Autoimmune manifestations and the discovery of autoreactive T cells and pathogenic autoantibodies in MCD patients would support it being an autoimmune disorder [1, 3]. However, it has been widely recognized that CMML is complicated with various types of autoimmune disorders. From these results, it was highly likely that Castleman disease is the underlying disease which triggers CMML for immune factors. Success in the setting of autoimmune dysfunction and thrombocytopenia has resulted in off-label use of the agents for patients with CMML, which verify our assumption in some ways. Alternatively, a study on therapy-related CMML concludes that chemotherapy and radiation are risk factors for the development of CMML. Therefore, we could not rule out in this case that chemotherapy is a risk factor for the development of CMML.

In summary, we report a rare case of MCD subsequently founding to have CMML, who responded with repeated long-lasting remissions to prednisone and decitabine therapy. Moreover, the findings in our study suggest a causal association between MCD and subsequent development of CMML for chronic inflammation/autoimmunity. The intrinsic immune pathogenesis of this unusual phenomenon remains to be further investigated.
